# Identifying and exploiting gene-pathway interactions from RNA-seq data for binary phenotype

**DOI:** 10.1186/s12863-019-0739-7

**Published:** 2019-03-19

**Authors:** Fang Shao, Yaqi Wang, Yang Zhao, Sheng Yang

**Affiliations:** 1 Department of Biostatistics, School of Public Health, Nanjing Medical University, 101 Longmian Avenue, Nanjing, Jiangsu People’s Republic of China; 2 Department of Pharmacy Informatics, School of Science, China Pharmaceutical University, 24 Tongjia Xiang, Nanjing , Jiangsu People’s Republic of China

**Keywords:** Gene-pathway interaction, Garrote kernel machine, Variance component testing, Binary phenotype

## Abstract

**Background:**

RNA sequencing (RNA-seq) technology has identified multiple differentially expressed (DE) genes associated to complex disease, however, these genes only explain a modest part of variance. Omnigenic model assumes that disease may be driven by genes with indirect relevance to disease and be propagated by functional pathways. Here, we focus on identifying the interactions between the external genes and functional pathways, referring to gene-pathway interactions (GPIs). Specifically, relying on the relationship between the garrote kernel machine (GKM) and variance component test and permutations for the empirical distributions of score statistics, we propose an efficient analysis procedure as Permutation based gEne-pAthway interaction identification in binary phenotype (PEA).

**Results:**

Various simulations show that PEA has well-calibrated type I error rates and higher power than the traditional likelihood ratio test (LRT). In addition, we perform the gene set enrichment algorithms and PEA to identifying the GPIs from a pan-cancer data (GES68086). These GPIs and genes possibly further illustrate the potential etiology of cancers, most of which are identified and some external genes and significant pathways are consistent with previous studies.

**Conclusions:**

PEA is an efficient tool for identifying the GPIs from RNA-seq data. It can be further extended to identify the interactions between one variable and one functional set of other omics data for binary phenotypes.

**Electronic supplementary material:**

The online version of this article (10.1186/s12863-019-0739-7) contains supplementary material, which is available to authorized users.

## Background

RNA sequencing (RNA-seq) technology has identified amounts of significant genes and given some evidence for the diagnosis and treatment of complex disease, especially cancers [1, 2]. Most of existing statistical methods focus on identifying the differentially expressed (DE) genes and heritability estimation by the RNA-seq count data [3–7]. However, with the assumption that only minority of genes associate with phenotypes, these models inevitably lose the regulation information from DE genes, thus are hard to elucidate the etiology and mechanism [8–10]. Systematic characterization of the biological function of genes represents an important step for investigating the molecular mechanisms underlying the identified disease associations. Enrichment analysis methods are based on different ideas: some only including genes participating in pathways and some considering the regulations between genes in networks [11–14].

Furthermore, omnigenic model assumes that disease may be driven by genes with indirect relevance to disease and be propagated by functional pathways. These external genes may cause the disease by distantly regulating significant pathways and they may explain most heritability [15]. For transcriptome data, one common sense is that the core gene effects can be understood by their interactions within underlying pathways or any expressed gene [16, 17]. As a result, identifying the interactions between external genes and significant pathways (GPIs) holds for further understanding of etiology and improving the prediction ability [18].

Considering the potential importance of interactions in defining the genetic architecture of complex traits and inefficiency of traditional methods in high-dimensional data, emerging statistical methods have been implemented to identify interactions with low calculation resources [19]. Different algorithms use different ideas, such as set tests [20, 21] and searching algorithms (exhaustive searching and prioritization based on the gene set) [22, 23]. Due to lacking confidence and biological priority and high-dimensional searching spaces, these methods may lose power.

Moreover, the omnibus test is widely used to identify the sets from both single and multiple levels, even from different datasets [24–29]. The joint test is more efficient and scalable because of low computational consumption, reduction of the degree of freedom and no estimation of variance components. On the other hand, kernel-based methods have been proposed to estimate association of genetic variants with complex traits [20, 28, 30–32]. A general kernel machine method can account for complex nonlinear genes and interactions effects. Though the application of kernel-based methods in genome wide association studies (GWASs) has been reported in the literature, our method applies the idea to identify GPIs of the transcriptome data [32, 33].

Here, noting the similarity between the mixed model and kernel function, we develop a statistical test to identify the GPIs for binary phenotypes. The model possibly solves the two challenges. First, our model is testing the GPIs in the binary phenotype framework. To do so, we firstly use two enrichment analysis of RNA-seq data, including gene set enrichment analysis (GSEA), DAVID and MinePath [11–13]. Second, the model is quite similar to the garrote kernel machine (GKM), but the parameter estimation procedure is quite different [20]. We refer to the statistical method as the **P**ermutation based g**E**ne-p**A**thway interaction identification in binary phenotypes (PEA). We provide a method overview of PEA, including the parameter estimation and hypothesis testing. Extensive simulations show that compared to the traditional likelihood ratio test (LRT) for generalized linear models, PEA has higher areas under curve (AUCs) with controllable type I error rates. In addition, the parameter estimation is more accurate. We apply our method to analyze platelet RNA-seq data from a case-control study (GSE68086) [1]. PEA can also be applied to analyze other interactions in binary phenotypes, such as pathway-environment interactions. PEA is implemented as a Rcpp function, freely available at https://github.com/biostat0903/RNAseq-Data-Analysis.

## Methods

### Model

**Fig. 1:**
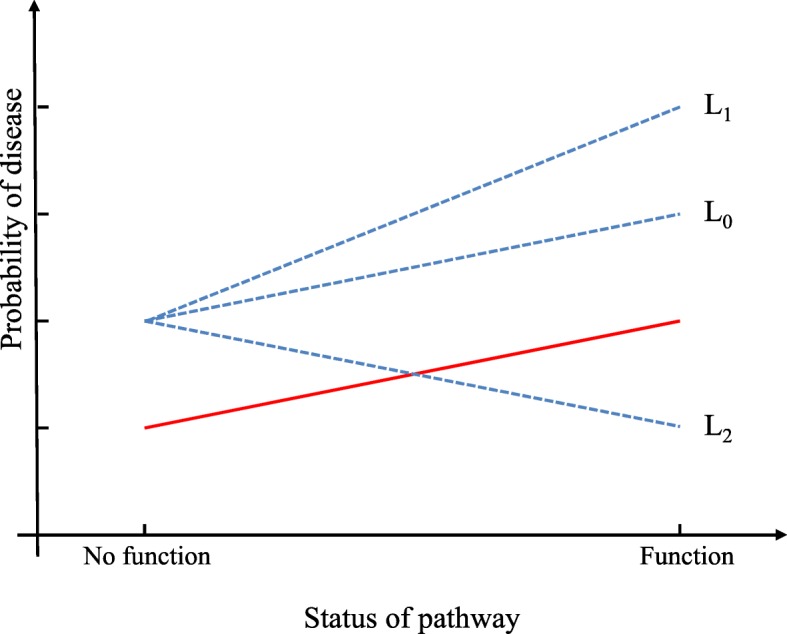
The illustration of GPI in binary phenotypes. The red line represents the effect of the significant pathway. The three black dot lines represent three situations: positive interaction (L1), no interaction (L0) and negative interaction (L2)

We model GPIs in binary phenotypes as following:

1 $$ \mathrm{logit}\left(P\left(\mathbf{y}=1\right)\right)=\mathbf{X}{\boldsymbol{\upbeta}}_{\boldsymbol{C}}+\sum \limits_{j=1}^P{\mathbf{P}}_j{{\boldsymbol{\upbeta}}_{\boldsymbol{P}}}_j+\mathbf{G}{\beta}_{\mathbf{G}}+\sum \limits_{j=1}^P\left({\mathbf{P}}_j\bullet \mathbf{G}\right){{\boldsymbol{\upxi}}_{\mathbf{G}}}_j $$

where **y** indicates the binary phenotypes (*i* = 1, … , *N*), **X**, an *N* × *m* matrix, is supposed as the covariates, **P**, an *N* × *P* matrix, is assumed as the expression levels in a significant pathway, which can be calculated from some gene enrichment analyses, **P**_*j*_ ***∙*** **G** indicate the GPIs (Fig. 1). We also suppose **γ** = *P*(**y** = 1). **β**_***C***_, **β**_***P***_, *β*_***G***_ and **ξ**_***G***_ are the coefficients of the covariates, functional pathway genes, external gene and GPIs, respectively.

LRT based on chi-squared statistics is a traditional method for testing of interactions for generalized linear models. The chi-squared statistic is the multiplication of − 2 by the logarithm of the ratio of likelihood of the full model and that of the model without interactions. Unfortunately, as the high-dimensional and complicated relation of the variables, traditional methods are always inefficient.

### Garrote kernel machine (GKM)

A kernel function is suitable to suggest the complicated relationship, including both linear and nonlinear relations, between genes and phenotypes. Here, we extend the linear GKM for the binary phenotype to identify the GPIs, although many other kernel functions can be selected. The kernel function is shown as following:

2 $$ \mathbf{K}\left({\mathbf{Z}}_k,{\mathbf{Z}}_l\right)=\left(1+{\delta \mathbf{G}}_k{\mathbf{G}}_{\boldsymbol{l}}\right)\left(1+{\mathbf{P}}_k{\mathbf{P}}_l\right) $$

where **Z**_*k*_ = (**G**_*k*_, **P**_*k*_), **K**(**Z**_*k*_, **Z**_*l*_) is the kernel matrix of *k*th and *l*th individuals. We then test for the effect of the GPIs by considering the null hypothesis *H*_0_ : *δ* = 0.

### Parameter estimation

With the kernel function, the Eq. (1) can be rewritten as a semi-parametric model as follows:

3 $$ \mathrm{logit}\left(P\left(\mathbf{y}=1\right)\right)=\mathbf{X}{\boldsymbol{\upbeta}}_{\boldsymbol{C}}+\mathbf{h} $$

where **h** = (h_1_, h_2_,  … , h_*N*_)^*T*^ is an unknown centered smooth function vector. **h** can be parameterized for different forms of GPIs, such as the Gaussian kernel and *d* th ploynomial kernel. As the similarity between the semi-parametric model and mixed effect model, the **h** can be assumed as the random effects following a multivariate normal distribution $\mathcal{N}\left(0,\tau \mathbf{K}\left(\updelta \right)\right)$. The relationship between the unknown function and the kernel function is as follows:

4 $$ {h}_i=h\left({\mathbf{Z}}_i\right)=\sum \limits_{l=1}^N{\boldsymbol{\upalpha}}_i\mathbf{K}\left({\mathbf{Z}}_i,{\mathbf{Z}}_l\right)={\kappa}_i^T\boldsymbol{\upalpha} $$

where ${\kappa}_i^T=\left(\mathbf{K}\left({\mathbf{Z}}_i,{\mathbf{Z}}_1\right),\mathbf{K}\left({\mathbf{Z}}_i,{\mathbf{Z}}_2\right),\dots, \mathbf{K}\left({\mathbf{Z}}_i,{\mathbf{Z}}_N\right)\right)$ and **α** is an unknown scale parameter vector.

Integrating the kernel function and logistic regression, the log-likelihood function is as following:

$$ {L}_{ML}=P\left(\boldsymbol{y}|{\boldsymbol{\upbeta}}_{\boldsymbol{C}},\mathbf{h}\right)=\sum \limits_{i=1}^N\left\{{\mathbf{y}}_i\left({\mathbf{X}}_i{\boldsymbol{\upbeta}}_{\boldsymbol{C}}+{\kappa}_i^T\boldsymbol{\upalpha} \right)-\mathit{\log}\left[1+\mathit{\exp}\left({\mathbf{X}}_{\boldsymbol{i}}{\boldsymbol{\upbeta}}_{\boldsymbol{C}}+{\kappa}_i^T\boldsymbol{\upalpha} \right)\right]\right\}-\frac{\lambda }{2}{\boldsymbol{\upalpha}}^{\boldsymbol{T}}\mathbf{K}\boldsymbol{\upalpha } $$

Furthermore, as the loss of the degree of freedom due to the maximum likelihood estimation of fixed effects, the estimate of the variance component *τ* is obtained by optimizing the restricted maximum log-likelihood (REML) function as following:

$$ {L}_{REML}\approx -\frac{1}{2}\left(\mathit{\log}\left|\mathbf{V}\right|+\mathit{\log}\left|{\mathbf{X}}^{\boldsymbol{T}}\mathbf{VX}\right|+{\left(\overset{\sim }{\mathbf{y}}-\mathbf{X}{\boldsymbol{\upbeta}}_{\boldsymbol{C}}\right)}^T{\mathbf{V}}^{-\mathbf{1}}\left(\overset{\sim }{\mathbf{y}}-\mathbf{X}{\boldsymbol{\upbeta}}_{\boldsymbol{C}}\right)\right) $$

where $\overset{\sim }{\mathbf{y}}=\mathbf{X}{\boldsymbol{\upbeta}}_{\boldsymbol{C}}+\mathbf{K}\boldsymbol{\upalpha } +{\mathbf{D}}^{-\mathbf{1}}\left(\mathbf{y}-\boldsymbol{\upgamma} \right)$ and **V = D**^***−*1**^ ***+*** *τ***K**. *τ* is estimated by the Newton-Raphson algorithm with damping factor ω = 0.5. The iteration formula is as following:

$$ {\tau}^{\left(t+1\right)}={\tau}^{(t)}-{\omega}^t\frac{{\left(\overset{\sim }{\mathbf{y}}-\mathbf{X}{\boldsymbol{\upbeta}}_{\boldsymbol{C}}\right)}^T{\mathbf{V}}^{-\mathbf{1}}{\mathbf{KV}}^{-\mathbf{1}}\left(\overset{\sim }{\mathbf{y}}-\mathbf{X}{\boldsymbol{\upbeta}}_{\boldsymbol{C}}\right)- tr\left(\mathbf{QK}\right)}{tr\left(\mathbf{QK}\mathbf{QK}\right)/2} $$

where **Q** = **V**^−**1**^ − **V**^−**1**^**X**(**X**^***T***^**V**^−**1**^**X**)^−**1**^**X**^***T***^**V**^−**1**^ and *t* is the iteration time.

**α** and **β**_***C***_ is estimated by the Newton-Raphson algorithm with normal equations as following:

5 $$ \left[\begin{array}{cc}{\mathbf{X}}^{\boldsymbol{T}}{\mathbf{D}}^{(t)}\mathbf{X}& {\mathbf{X}}^{\boldsymbol{T}}{\mathbf{D}}^{(t)}\mathbf{K}\\ {}{\mathbf{D}}^{(t)}\mathbf{X}& {\tau}^{-1}\boldsymbol{I}+{\mathbf{D}}^{(t)}\mathbf{K}\end{array}\right]\left[\begin{array}{c}{\beta}_{\boldsymbol{C}}^{\left(t+\mathbf{1}\right)}\\ {}{\boldsymbol{\alpha}}^{\left(t+\mathbf{1}\right)}\end{array}\right]=\left[\begin{array}{c}{\mathbf{X}}^{\boldsymbol{T}}{\mathbf{D}}^{(t)}{\overset{\sim }{\mathbf{y}}}^{(t)}\\ {}{\mathbf{D}}^{(t)}{\overset{\sim }{\mathbf{y}}}^{(t)}\end{array}\right] $$

### Hypothesis testing

As the similarity between the mixed model and semi-parametric model, we propose a score test statistic *U* to test *δ*:

$$ U=\frac{1}{2}{\left(\overset{\sim }{\mathbf{y}}-\mathbf{X}{\boldsymbol{\upbeta}}_{\boldsymbol{C}}\right)}^T{\mathbf{V}}^{-\mathbf{1}}\frac{\partial \mathbf{V}}{\mathrm{\partial \updelta }}{\mathbf{V}}^{-\mathbf{1}}\left(\overset{\sim }{\mathbf{y}}-\mathbf{X}{\boldsymbol{\upbeta}}_{\boldsymbol{C}}\right) $$

As the $\frac{\partial \mathbf{V}}{\mathrm{\partial \updelta }}$ is not a semi-definite matrix, *U* is not identically greater than zero. Its distribution is hard to use the mixed chi-squared distribution to approximate. Therefore, we propose a permutation test to obtain the empirical distribution of *U*. Since the null hypothesis is *δ* = 0, we permute the expression level of the external gene and calculate the test statistic without re-estimation of the **β**_***C***_, **α** and *τ*. The computation consumption is not large, although we use a permutation test.

### Simulation

Compared to the traditional LRT, simulations investigate the statistical property of *U* and the estimation of **β**_***C***_. Type I error rates and AUCs show the statistical property of statistics. Means and standard errors indicate the estimation property. The expressed levels of the functional pathway are generated from correlated uniform distributions by a Copula function, and that of the external gene is generated from a uniform distribution ($\mathcal{U}\left(0,1\right)$). We study five parameters in simulations: sample size (*N*), number of genes in the functional pathway (*v*), correlation between genes in the pathway (*c*), proportion of interaction genes (*p*) and odds ratio (OR) of the external gene (*s*). Details are shown in the Table 1. The interaction function *h* for the *i* th individual is defined by a function *g* as following:

$$ g\left({\mathbf{Z}}_i\right)=g\left({\mathbf{Z}}_{i1},{\mathbf{Z}}_{i1},\dots, {\mathbf{Z}}_{i1}\ \right)=1+\sum \limits_{j=1}^P{\mathbf{P}}_{ij}{{\boldsymbol{\upbeta}}_{\boldsymbol{P}}}_{ij}+{\mathbf{G}}_i{\beta}_{\mathbf{G}}+\sum \limits_{j=1}^P\left({\mathbf{P}}_{ij}\bullet {\mathbf{G}}_i\right){{\boldsymbol{\upxi}}_{\mathbf{G}}}_{ij} $$

**Table 1:** Parameter settings used for the simulation data

Parameters	Label	Settings
sample size	*N*	100, 200
number of genes in potential pathway	*v*	10,20, 30
correlation between genes in pathway	*c*	0, 0.25, 0.5
proportion of interaction genes	*p*	0.8, 1.0
OR of core gene	*s*	0, 1.5, 1.8, 2.0
OR of interactions		exp(*s*)/2
OR of genes in potential pathway		1.2

*h* can be defined as linear and non-linear settings: *h*(**Z**_*i*_) = *g*(**Z**_*i*_) and *h*(**Z**_*i*_) = *g*(**Z**_*i*_)^2^.

We simulate 1000 times at the null hypothesis (*s* = 0) and 100 times at the alternative hypothesis (*s* ≠ 0). Combing the 900 *P* values randomly selected from the null and 100 *P* values at the alternative, we can calculate the AUC. For each loop, we permute the label 50,000 times to obtain robust results.

### Real data analysis

We use our method to analyze RNA-seq data of 283 platelet samples (55 healthy donors and 228 tumor samples) [1]. The tumor data is collected from six different cancers, including breast cancer (BrCa, *n* = 35), non-small cell lung carcinoma (NSCLC, *n* = 60), glioblastoma (GBM, *n* = 39), colorectal cancer (CRC, *n* = 41), pancreatic cancer (PAAD, *n* = 35) and hepatobiliary cancer (HBC, *n* = 14). The covariates are gender and age. We delete the individuals with missing data, and the final sample size is 274. The tumor samples are regarded as cases to perform pan-cancer analysis [1].

qTo ensure validity and reasonability, we follow the data processing steps of the original paper. First, we exclude the genes with total counts less than 5 and with logarithmic counts per million (LogCPM) less than 3. We only select 5003 genes for subsequent analysis. Second, we use the trimmed mean of M-value (TMM) normalization data for following analysis. Final, edgeR with tagwise and common dispersion is applied to select the external genes with the threshold of false discovery rate (FDR) < 0.001. We can obtain 2500 DE genes, including 1231 de-regulated and 1269 up-regulated genes.

After the filtering and normalizing, we perform three gene enrichment methods to obtain the functional pathways, including DAVID, GSEA and MinePath. DAVID and GSEA are performed by both Gene Ontology (GO) dataset and Kyoto Encyclopedia of Genes and Genomes (KEGG) dataset [34, 35]. MinePath is only based on KEGG dataset. The significant pathways are defined with FDR < 0.001 of DAVID, FDR < 0.25 of GSEA and FDR < 0.05 of MinePath. *biomaRt* package is used to map Ensembl ID to corresponding Entez ID and gene symbol [36]. For the PEA model, we normalize RNA-Seq data to the numbers between 0 and 1.

## Results

### Simulations

**Fig. 2:**
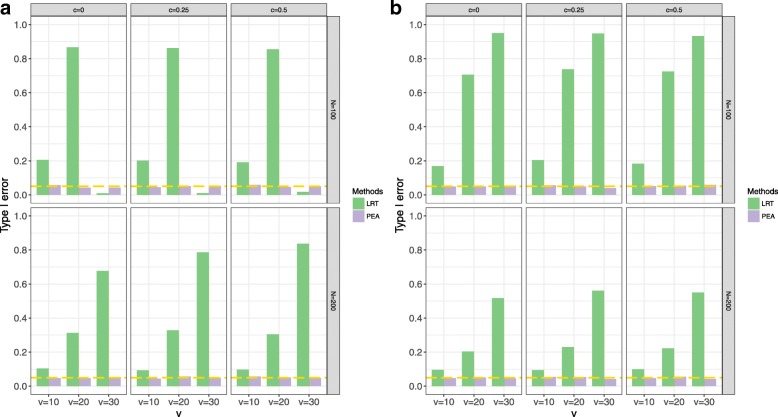
Type I error rates of PEA and traditional LRT in different interaction function settings, *N*, *v* and *c*. **a** linear interaction function settings; **b** nonlinear interaction function settings

**Fig. 3:**
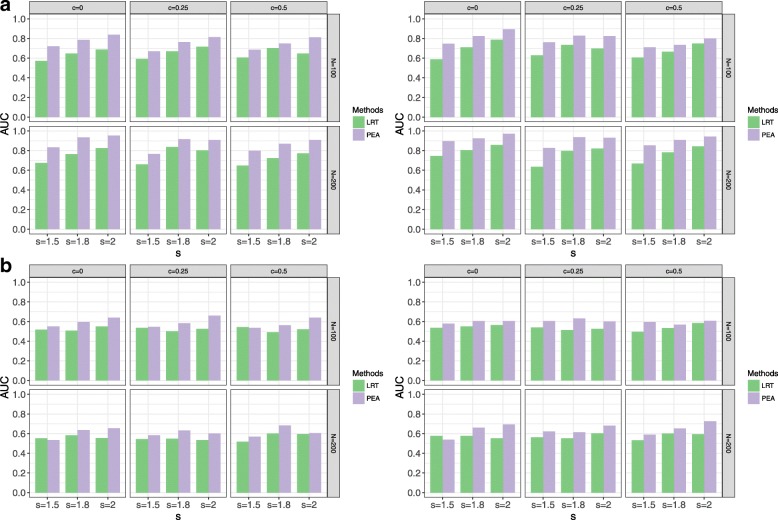
AUCs of PEA and traditional LRT in different interaction function settings, *N*, *c, s*, *p* and *v* = 10. **a** linear interaction function settings with *p* = 0.8 (left) and *p* = 1 (right); **b** nonlinear interaction function settings with *p* = 0.8 (left) and *p* = 1 (right)

**Fig. 4:**
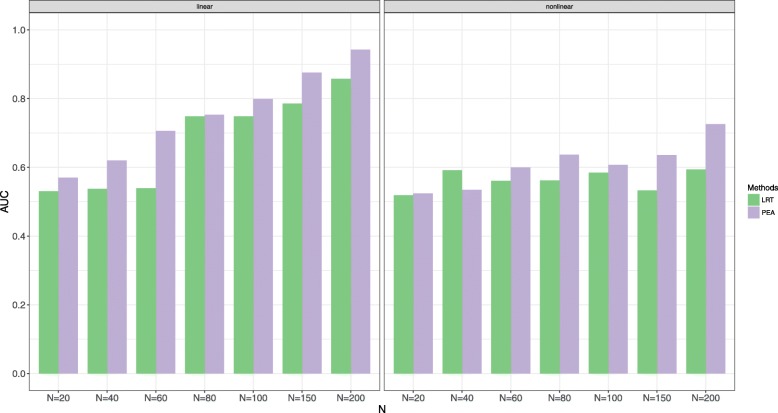
AUCs of PEA and traditional LRT in different sample size settings of different interaction function settings with *c* = 0.5, *s* = 2, *p* = 1 and *v* = 10

**Fig. 5:**
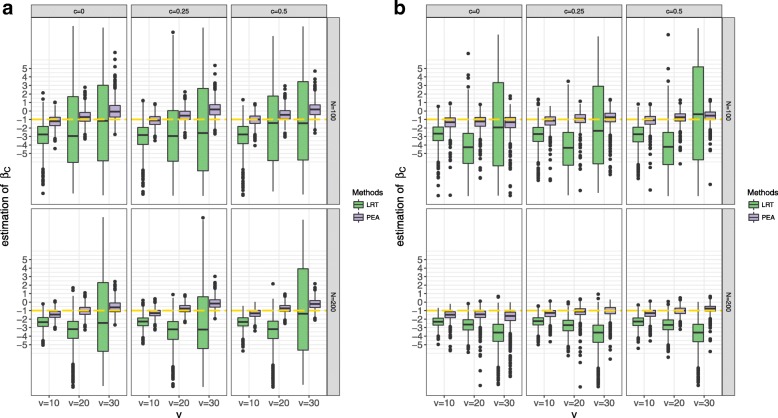
Estimation of **β**_***C***_ of PEA and traditional LRT in different settings of different interaction function settings, *N*, *v* and *c*. **a** linear settings; **b** nonlinear settings

Simulations evaluate the performances of PEA and traditional LRT by type I error rates and AUCs. In the type I error simulations, we use four different settings: different interaction function settings, *N*, *v* and *c*. In the power simulations, we add *s* and *p*. For the type I error simulations, the response variable is not affected by the effects of the external gene and its interactions. All the results are shown in Figs. 2, 3, 4 and 5 and Additional files 1 and 2. The significant level is set to be 0.05.

As expected, PEA controls type I error rates in all parameter settings, but the traditional LRT fails. Especially, in the non-linear setting, the type I error rates of PEA are close to the significant level, which shows this method properly controls the type I error rates. When *v* = 30, the traditional LRT is inefficient because of the unbalance between the number of variables and the sample size setting.

As the traditional method is uncontrollable for type I error rates, AUC is used to evaluate the performances of the two methods in the alternative simulations. When *v* = 10, it is clear that PEA is better than LRT in each setting, especially for the non-linear scenarios. When *v* = 20 and *v* = 30, the traditional LRT is better than PEA with the non-linear settings and *N* = 100. Increasing *s* and *N* improves power of PEA, but increasing *p* and *c* decreases power of PEA. The linear settings possibly lead to higher power. The minimum sample size is suggested as 100 by the different *N* settings.

### Real data analysis

After the enrichment analyses of DAVID and GSEA, we obtain 67 functional pathways (Additional file 3). We identify the GPIs between 2500 DE genes and four biological pathways: RNA processing (GO:0006396, size = 285), RNA splicing (GO:0008380, size = 127), cytosolic ribosome (GO:0022626, size = 80) and cytoplasmic translation (GO:0002181, size = 25). We identified 116, 109, 89 and 124 significant external genes for the different pathways at the nominal level 0.05, respectively. After filtering by the FDR < 0.2, the significant gene numbers are 10, 17, 54 and 83.

After the enrichment analyses of DAVID, GSEA and MinePath, we obtain 2 functional pathways (Additional file 4). We identify the GPIs between 2500 DE genes and two biological pathways: Platelet activation (hsa04611, size = 70) and Fc gamma R-mediated phagocytosis (hsa04666, size = 49). We identified 468 and 475 significant external genes for the two pathways at nominal level 0.05, respectively. After filtering by the FDR < 0.2, the significant gene numbers are 119 and 120.

As the different sizes of the four pathways, the power is different for the four pathways. The result of FDR controlling is similar to that of the simulations. Interestingly, we define multiple same genes for the four GO pathways, such as *TUBB*, *BPI* and *CA1*. Two KEGG pathways also interact with 11 common genes, such as *SDCBP*, *TRAT1* and *FABP4.* The summary of the result is shown in Table 2.

**Table 2:** Summary of the external genes for the selected pathways

Database	Pathway	Size	Top genes^a^
GO	RNA processing	285	*TUBB, H3F3B, SDCBP, NT5C2, FKBP5*
GO	RNA splicing	127	*TUBB, IL1R2, H3F3B, IFNGR1, PCSK7*
GO	cytosolic ribosome	80	*TUBB*, *CAMP, SAMM50, PSMD14, CHD8*
GO	Cytoplasmic translation	25	*TUBB, SDCBP, H3F3B, CMAHP, SLC44A2*
KEGG	Platelet activation	70	*STX7, PCNP, SDCBP, LEF1, TMEM140*
KEGG	Fc gamma R-mediated phagocytosis	49	*STX7, PCNP, CGRRF1, SHOC2, EFTUD2*

^a^Top five external genes for the significant pathway

## Discussion

Here, we present an effective semi-parametric generalized linear model, together with a computationally efficient parameter estimation method and software implementation of PEA, for identifying potential GPIs of RNA-seq data in binary phenotypes. PEA models the complicated relationship between gene expression and traits using the kernel function. Because the kernel machine can be adaptive for both linear and nonlinear interactions, PEA controls type I error rates in the presence of individual relatedness, and PEA achieves higher power than the traditional method, LRT, across a range of settings. In addition, PEA is available to other interactions of different molecules, such as methylation and gene expression interactions, and biological or technical covariates interactions. We have demonstrated the benefits of PEA using both simulations and applications to recently published RNA-seq datasets.

While PEA is an extension of the GKM, we note that PEA exploits the GPIs in binary phenotypes and estimates the parameters using the damping Newton-Raphson algorithm. As most of medical studies are case-control design, PEA identifies the GPIs for the binary traits. For example, samples are collected from tumor tissue and normal tissue, along with some covariates, such as age, gender and so on. Two Newton-Raphson iterations accurately estimate the coefficients of covariates (Fig. 5). GKM estimates *τ* by the *optimx* function in R using the Nelder-Mead method, which is not suitable for PEA.

In the real data analysis, the result of PEA can support the assumption of omnigenic model. Although PEA goes beyond the scope of enrichment analysis, efficient enrichment analysis methods, such as MinePath, can essentially provide the robust and reliable pathways before conducting PEA. PEA identify not only some significant GPIs, but also the external genes for different pathways. Some significant genes are verified by multiple biological studies. For example, using the data from The Cancer Genome Atlas (TCGA), *TUBB* expression level influences the survival time of renal and liver cancer [37]. Kelly et al. demonstrate that the mutation of *TUBB* possibly cause the tumor cell growth and taxane resistance for the patients with NSCLC [38]. From the result of PEA, *TUBB* might be an important external gene, which associates cancers by the interactions with amount significant pathways.

Currently, despite the newly developed computationally efficient statistical testing method, applications of PEA can still be limited by its relatively heavy computational cost of permutations. Traditional permutation tests increase the computation consumption with the shuffling times, but the permutations of PEA are faster than the standard permutation test because of no parameter re-estimation in each shuffling. PEA can still take close to 7 h with 5 CPUs to analyze a dataset of the size of the GSE68086 which we considered here (274 individuals ~ 2500 genes for one pathway). Therefore, PEA will be used to analyze other datasets that have large sizes.

## Conclusions

PEA is an efficient and powerful statistical method for identifying the GPIs from RNA-seq data. It can be further extended to identify the interactions between one variable and one functional set of other omics data for binary phenotypes. Further work is needed to make its widely use in more high-dimensional genomics data analysis practice.

## Additional files

Additional file 1: AUCs of PEA and traditional LRT in different interaction function settings, *N*, *c, s*, *p* and *v* = 20. (A) linear interaction function settings with *p* = 0.8 (left) and *p* = 1 (right); (B) nonlinear interaction function settings with *p* = 0.8 (left) and *p* = 1 (right). (PDF 27 kb)

Additional file 2: AUCs of PEA and traditional LRT in different interaction function settings, *N*, *c, s*, *p* and *v* = 30. (A) linear interaction function settings with *p* = 0.8 (left) and *p* = 1 (right); (B) nonlinear interaction function settings with *p* = 0.8 (left) and *p* = 1 (right). (PDF 27 kb)

Additional file 3: The enriched GO pathways from both GSEA and DAVID. (XLSX 13 kb)

Additional file 4: The enriched KEGG pathways from three algorithms. (XLSX 9 kb)

## Abbreviations

AUC: Area under curve
BrCa: Breast cancer
CRC: Colorectal cancer
DE: Differentially expressed
FDR: False discovery rate
GBM: Glioblastoma
GKM: Garrote kernel machine
GO: Gene Ontology
GPI: Gene-pathway interaction
GSEA: Gene set enrichment analysis
GWAS: Genome wide association study
HBC: Hepatobiliary cancer
KEGG: Kyoto Encyclopedia of Genes and Genomes
LogCPM: Logarithmic counts per million
LRT: Likelihood ratio test
NSCLC: Non-small cell lung carcinoma
PAAD: Pancreatic cancer
PEA: Permutation based gEne-pAthway interaction identification for binary phenotypes
REML: Restricted maximum likelihood
RNA-seq: RNA sequencing
TCGA: The Cancer Genome Atlas
TMM: Trimmed mean of M-value
