# Supplementary figures and images for: Identifying and exploiting gene-pathway interactions from RNA-seq data for binary phenotype
Source: BMC Genet. 2019 Mar 19;20:36. doi: 10.1186/s12863-019-0739-7 (PMC6423879; doi:10.1186/s12863-019-0739-7)

**A**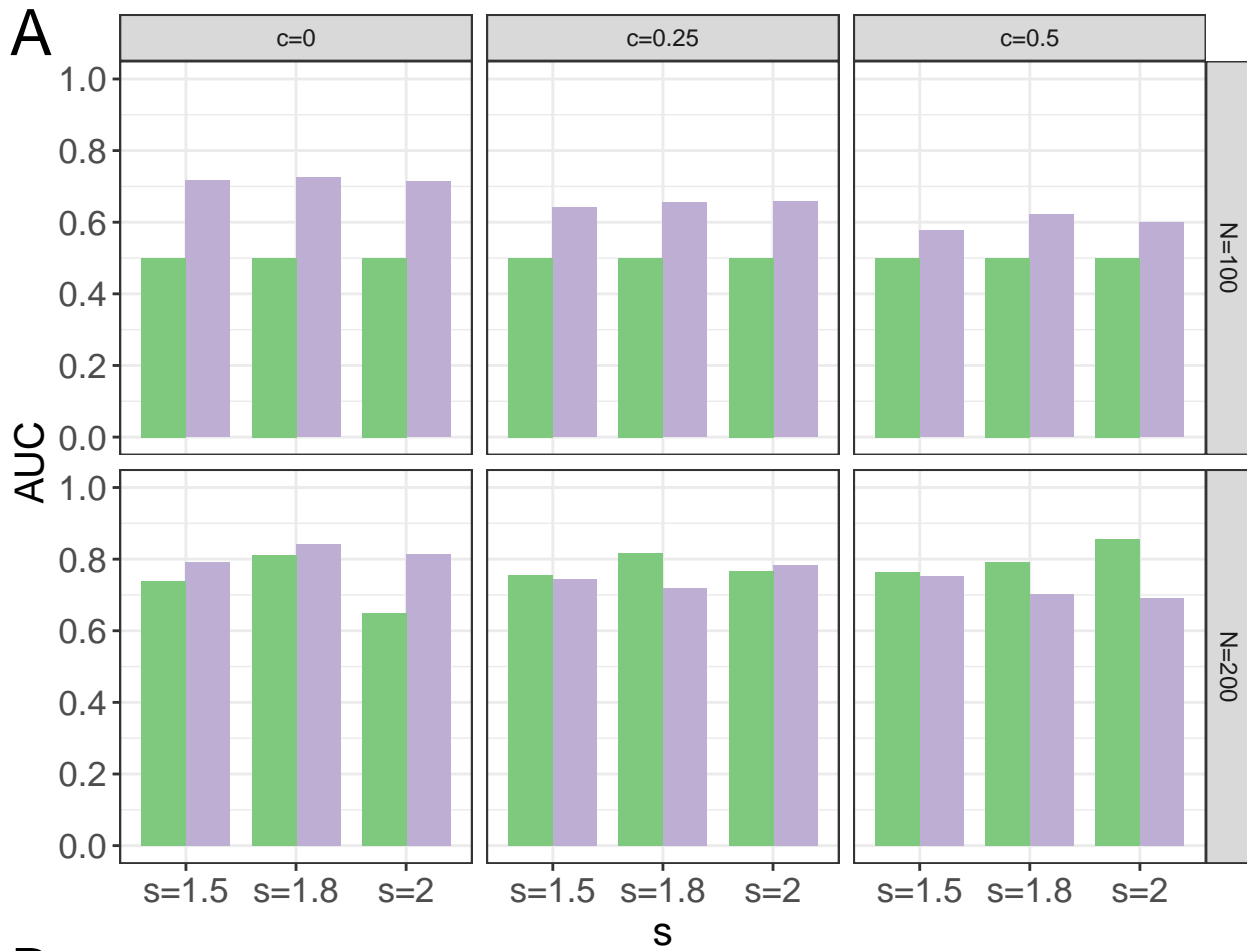

Methods

LRT

PEA

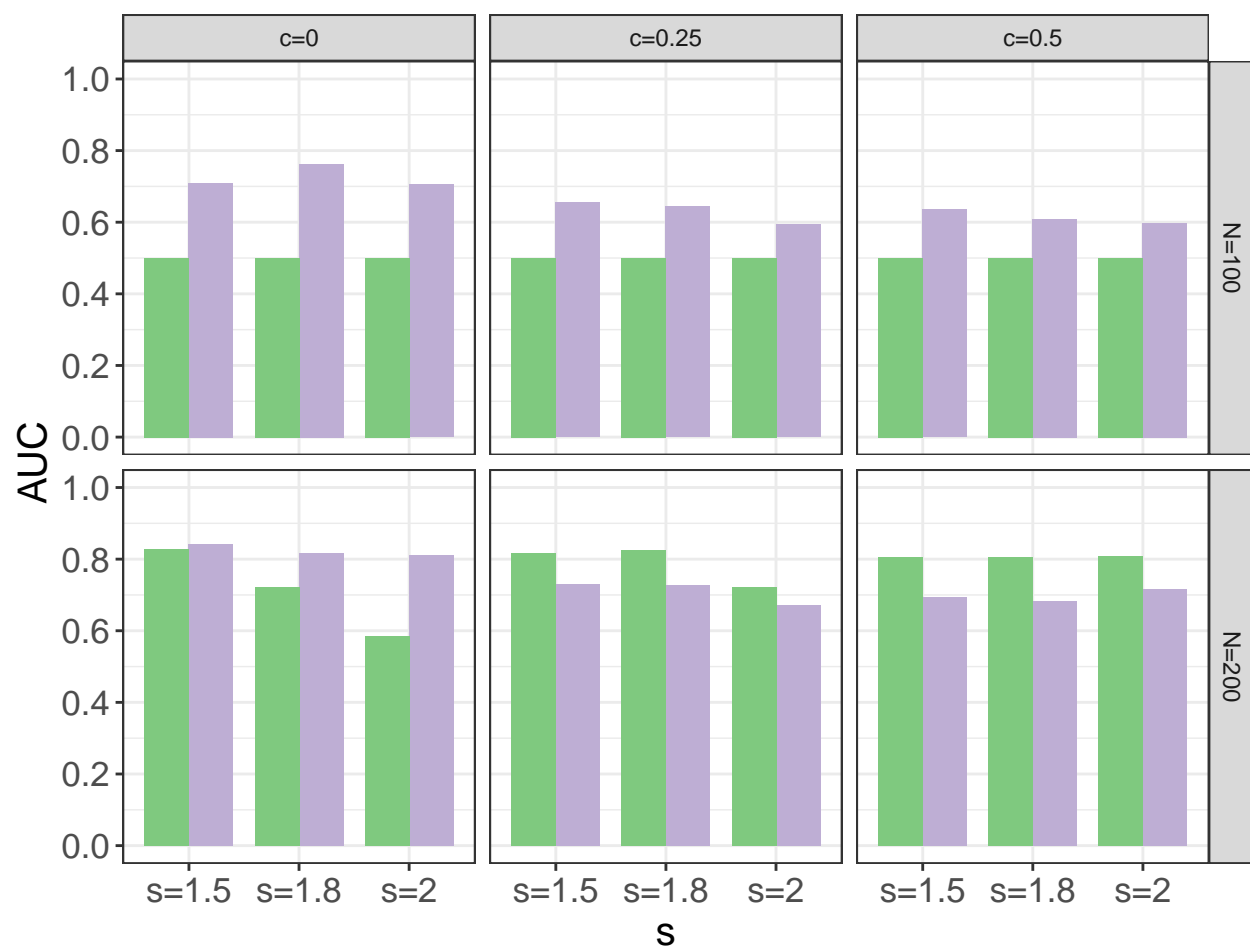

Methods

LRT

PEA

**B**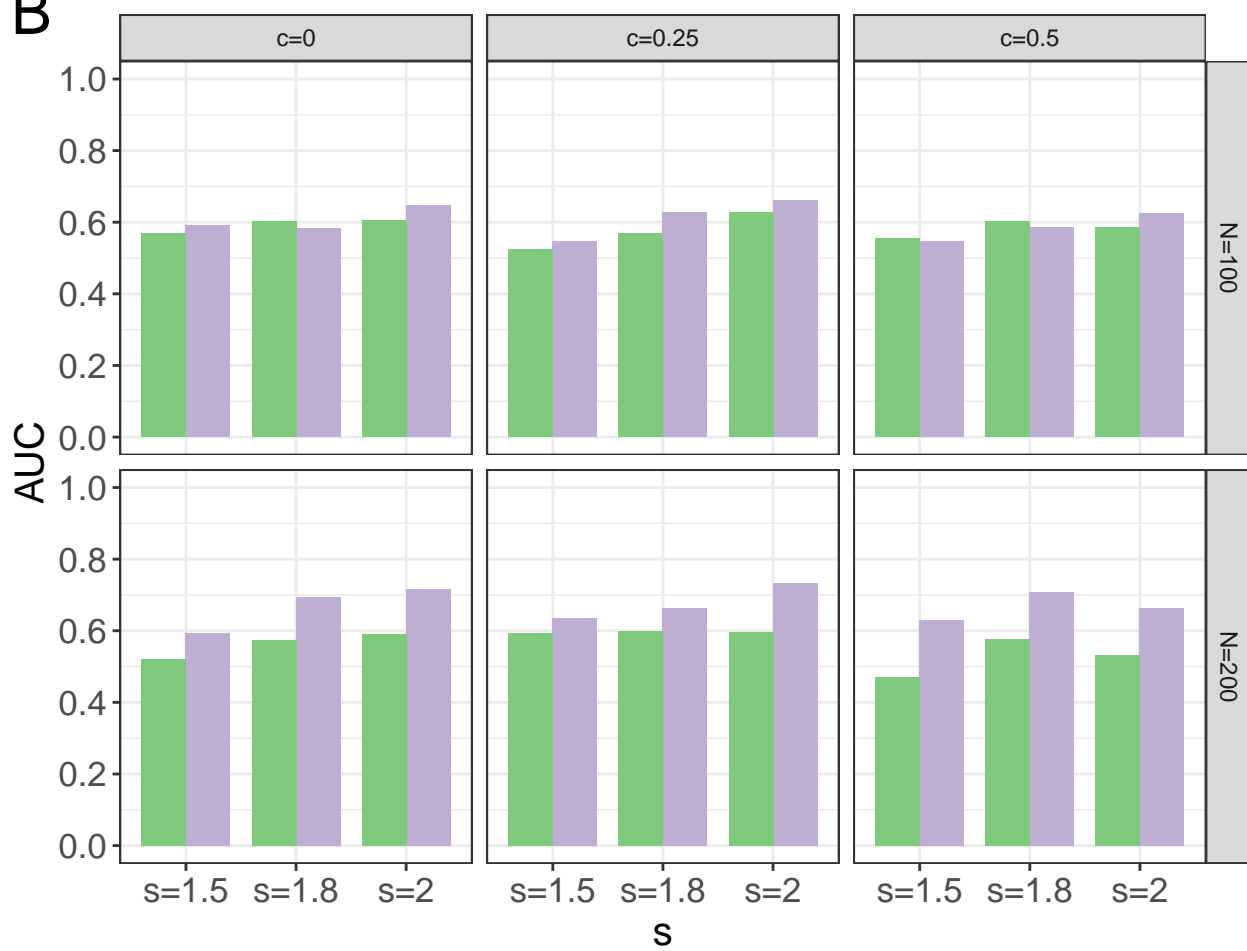

Methods

LRT

PEA

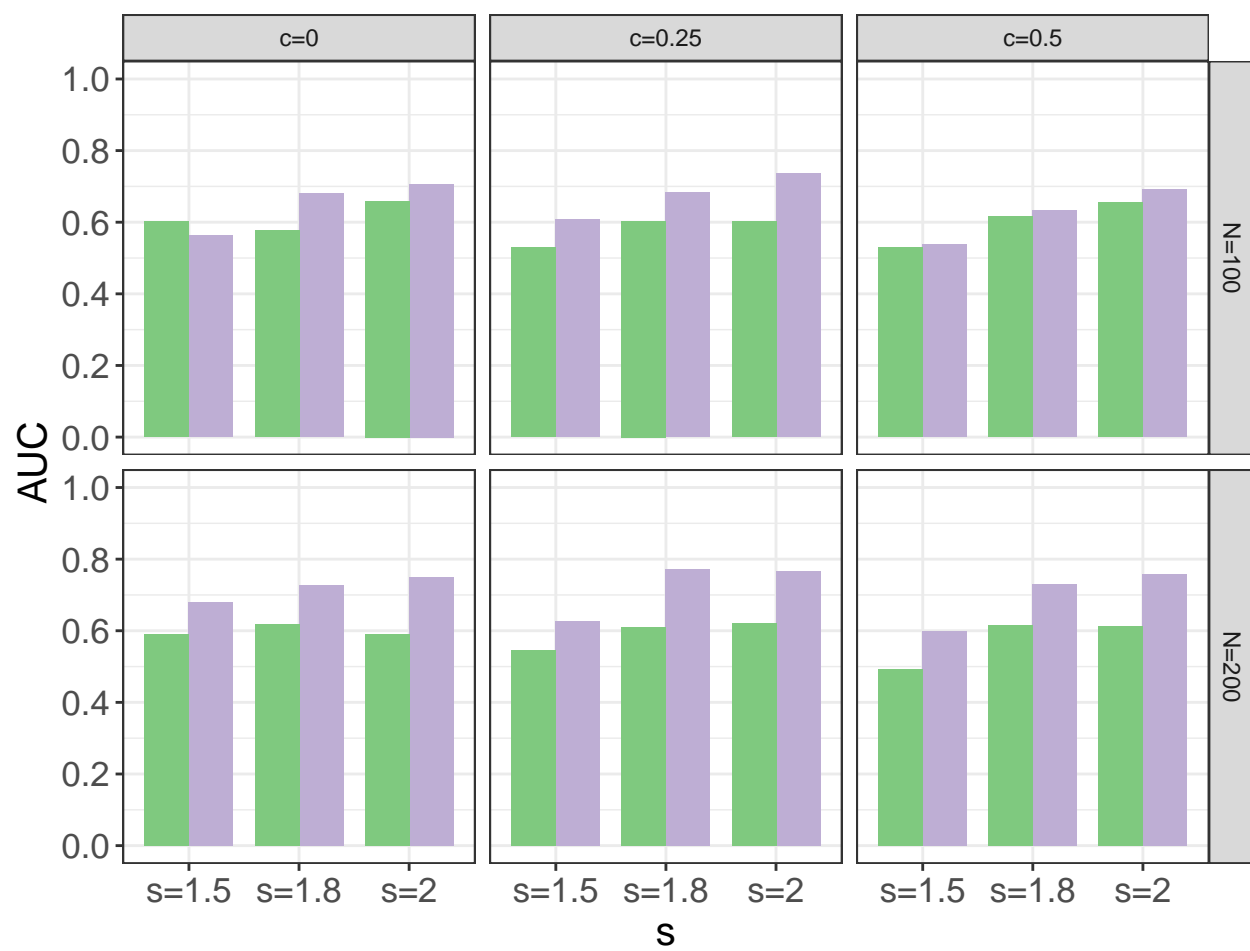

Methods

LRT

PEA

Supplement: Supplementary file 1 — AUCs of PEA and traditional LRT in different interaction function settings, N, c, s, p and v = 20. (A) linear interaction function settings with p = 0.8 (left) and p = 1 (right); (B) nonlinear interaction function settings with p = 0.8 (left) and p = 1 (right). (PDF 27 kb) [file 12863_2019_739_MOESM1_ESM.pdf]

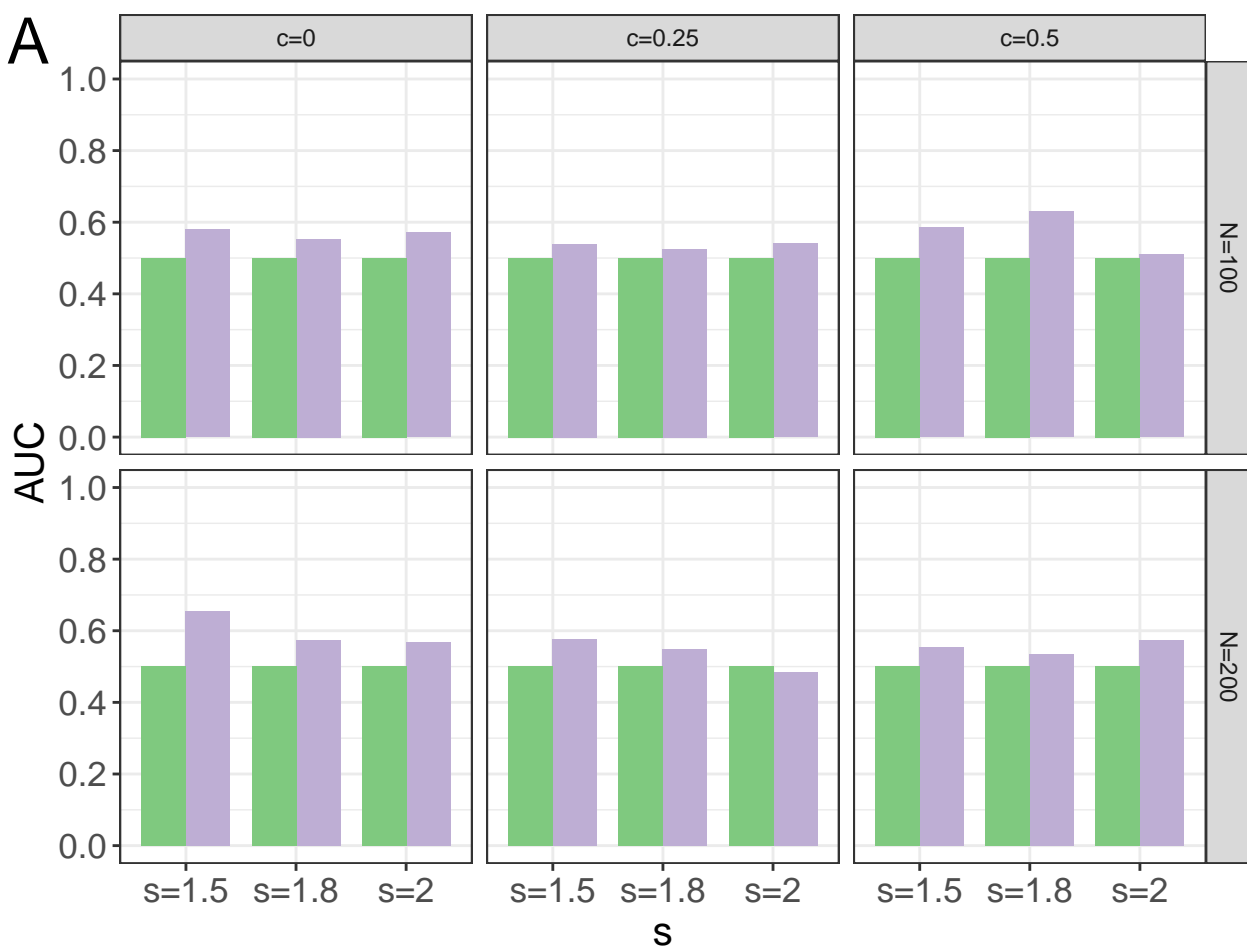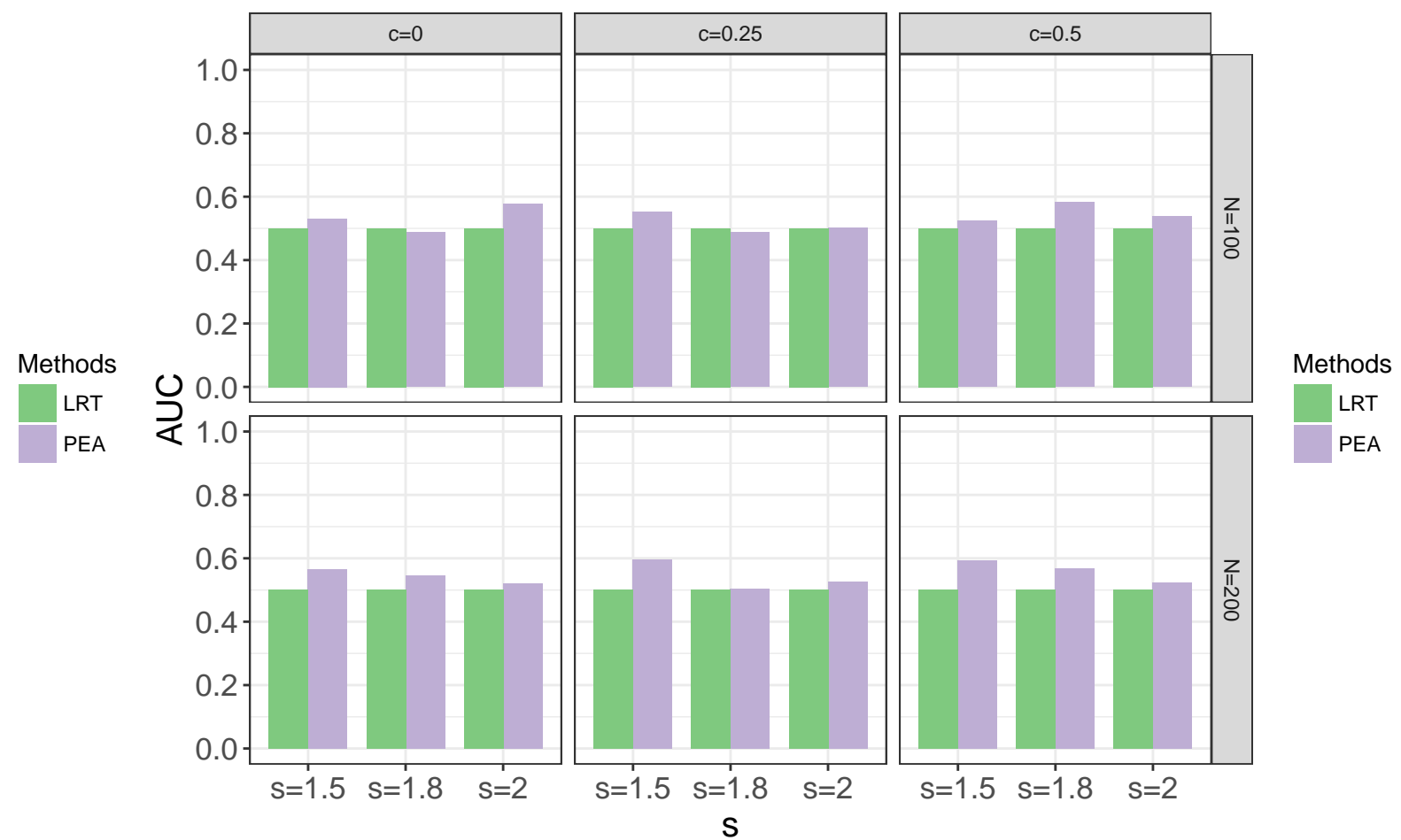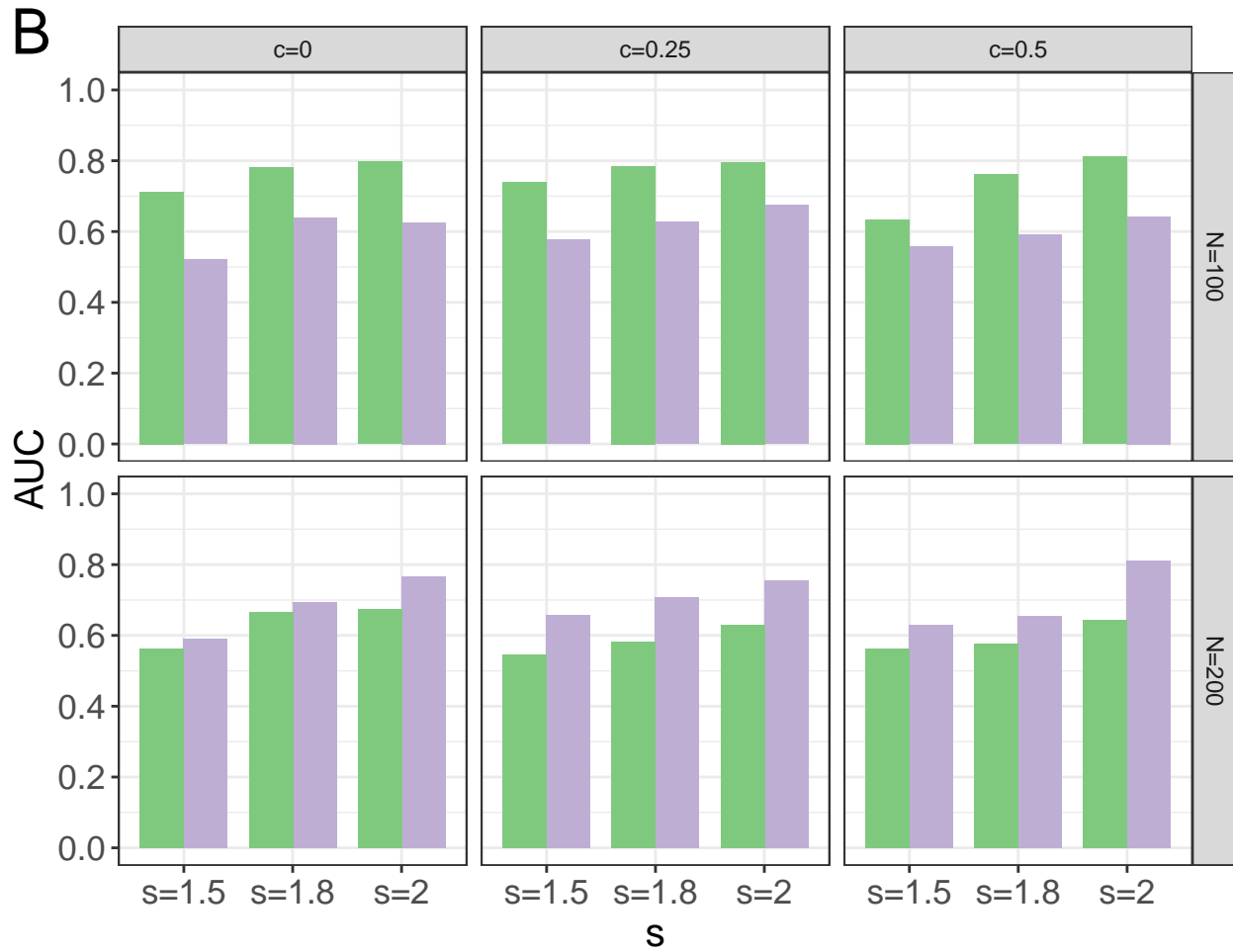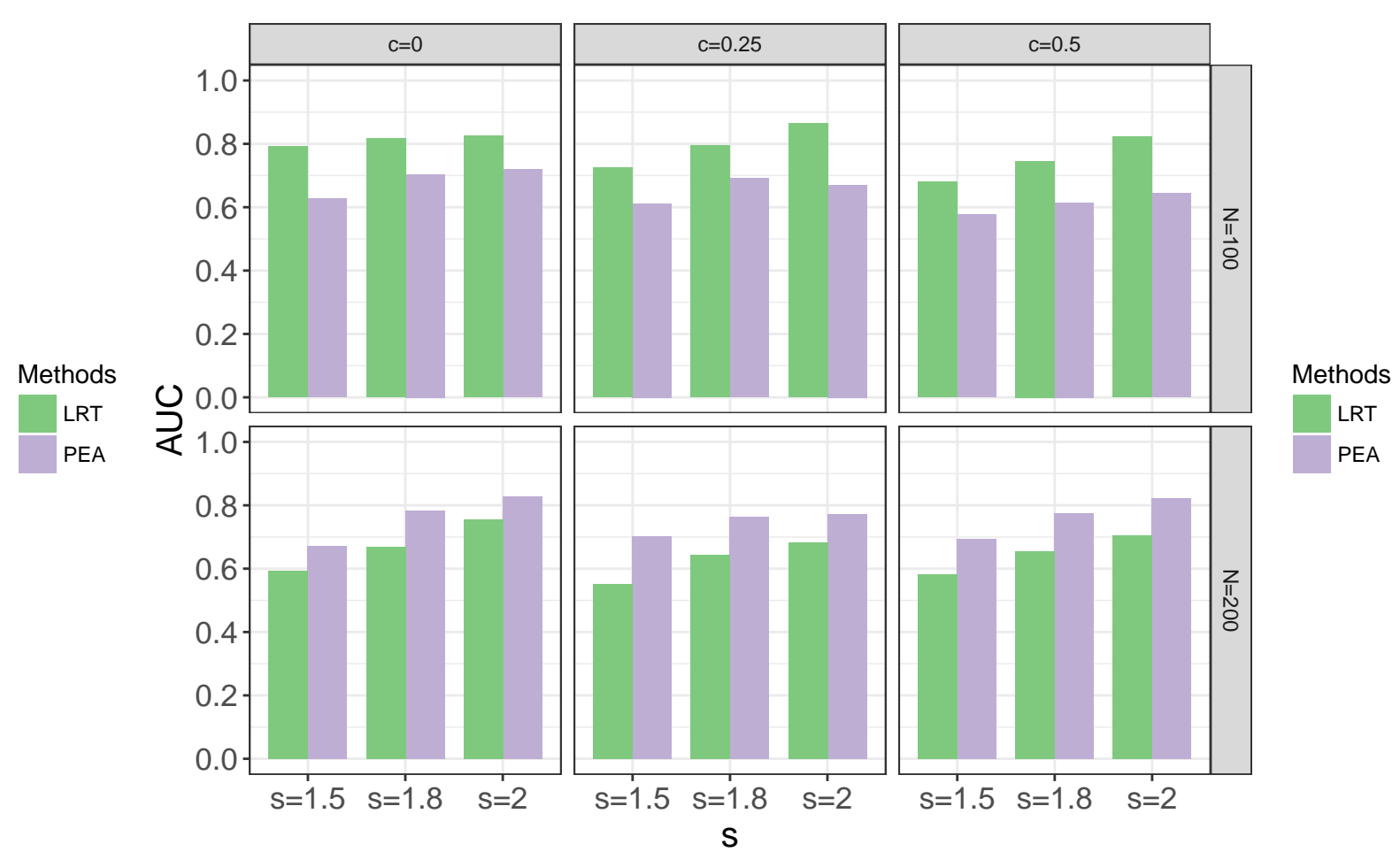

Supplement: Supplementary file 2 — AUCs of PEA and traditional LRT in different interaction function settings, N, c, s, p and v = 30. (A) linear interaction function settings with p = 0.8 (left) and p = 1 (right); (B) nonlinear interaction function settings with p = 0.8 (left) and p = 1 (right). (PDF 27 kb) [file 12863_2019_739_MOESM2_ESM.pdf]
